# Emergence Delirium in a 29-Year-Old Man following an Uneventful Appendectomy

**DOI:** 10.1155/2021/1338823

**Published:** 2021-09-25

**Authors:** Zhi Wang, Yong Yang, Yang Chen, Kai Lu, Bing Chen

**Affiliations:** ^1^Department of Anesthesia, Southwest Hospital, Army Military Medical University, Chongqing 400038, China; ^2^Department of Anesthesia, The Second Affiliated Hospital of Chongqing Medical University, Chongqing 400010, China

## Abstract

Emergence delirium (ED) is defined as the delirium that occurs during the transition from the sleep state to full consciousness. ED increases the risk for injury, self-extubation, hemorrhages, and prolonged hospitalization and occurs in patients of any age but most often in children and elderly patients. However, ED in young adults is rarely reported. We presented a case of typical ED occurring in a young healthy man following an uneventful appendectomy. The causes of ED can be classified as either predisposing or precipitating factors. In this case, the unnoticeable mental stress may be the predisposing factor and the sevoflurane maintenance of anesthesia may be the precipitating factor. ED occurs at any age of patient and in any minor surgery, and anesthesiologists should do some work to prevent it from happening.

## 1. Introduction

Postoperative delirium (POD) is an acute and fluctuating alteration of mental state of reduced awareness and disturbance of attention, typically occurring between 24 and 72 hours after surgery [1]. Emergence delirium (ED) is defined as the delirium that occurs during the transition from the sleep state to full consciousness [2]. ED is an adverse postoperative complication that occurs in patients of any age but most often in children with a variable incidence of 10–80% [3] and elderly patients between 10% and 15% [4]. ED increases the risk for injury, self-extubation, hemorrhages, and prolonged hospitalization [5]. The European Society of Anesthesiology evidence-based and consensus-based guideline on POD [6] states that the risk factors for POD include advanced age, combination of other systemic diseases (e.g., cerebrovascular including stroke, Parkinson's disease, depression, and anxiety disorders), alcohol-related disorders, preoperative fluid fasting and dehydration, electrolyte imbalance, drugs with anticholinergic effects, operation site, intraoperative bleeding, operation time, and postoperative pain. Factors contributing to pediatric ED are as follows: preschool age, psychological immaturity, preoperative anxiety, surgical categories (e.g., ear, nose, and throat surgery), inhalational maintenance of anesthesia, pain, and physiological condition (e.g., hypoglycemia and electrolyte imbalance) [3]. However, ED in young adults is poorly investigated. We presented a case of typical ED occurring in a young healthy man following an uneventful appendectomy and discussed the potential mechanisms.

## 2. Case Presentation

A 29-year-old man, 60 kg, body mass index 21.3 kg/m^2^, was presented for an emergency appendectomy. The acute right lower quadrant pain occurred a day before. The peripheral blood leukocyte counts (13.23 × 10^9^/L) and abdominal ultrasound examination indicated acute appendicitis, and other examinations were normal. He had a normal mental state, fair sleep and appetite, normal urination and defecation, and no change in body weight within half a year. He and his relatives had no history of drug abuse, alcohol or nicotine abuse, specific medication intake regularly, metabolic diseases, allergic reactions, treatment with psychiatric drugs, epilepsy, or neurological disorder.

No premedications were given. Upon arrival in the operating room, midazolam 2 mg and dexamethasone 10 mg were administered intravenously. Anesthesia was induced by intravenous injection of propofol 100 mg, sufentanil 20 *μ*g, and rocuronium 35 mg and maintained by sevoflurane inhalation and continuous infusion of remifentanil. A size #4 laryngeal mask airway was inserted for ventilation. The surgery took 2 hours to complete and was uneventful with no significant blood loss. No adverse events, such as hypoglycemia, electrolyte imbalance, hypoxemia, and hypercapnia, occurred during the procedure. A patient-controlled intravenous analgesia (PCIA) with sufentanil 100 *μ*g, dexmedetomidine 100 *μ*g, and butorphanol 5 mg was used for postoperative analgesia. To accelerate emergence, a flow rate of 8 L/min with 60% O_2_ was used to “wash out” sevoflurane after turning off the anesthesia vaporizer. About 5 min later, spontaneous breathing recovered, but his eyes were closed and he did not respond to anesthesiologist's commands. Neostigmine 2 mg and atropine 1 mg were intravenous injected to antagonize residual rocuronium, and flumazenil 0.3 mg was injected to reverse midazolam. After 5-minute assessment of the ventilation volume and rate and capnography, the patient was extubated but was still unresponsive, and he tightly closed his eyes and began to sob with no tears and shake his head continuously (Supplementary video 1). He looked very sad. This condition lasted approximately 2 to 3 minutes, followed by somnolence with eyes closed and continuous opening and closing of his jaw that lasted approximately 3 to 5 minutes (Supplementary video 2). These two situations were repeated continuously. Vital signs were stable, and oxygen saturation remained at 97% to 100% during this period. The patient was transferred to the postanesthesia care unit. His wife was invited to comfort the patient, but he was still unresponsive and had no alleviation of the symptoms. This patient completely self-recovered within 5 hours without drug intervention. After recovery, the patient could not recall what happened in the past several hours. However, he felt a voice was calling him and he could not wake up. Moreover, he denied having experienced any pain associated with the surgical procedure. After a detailed history review, the patient had a little worry about his occupation mobility in the past year, but that did not meet the diagnostic criteria for generalized anxiety disorder according to the 5th Edition of Diagnostic and Statistical Manual of Mental Disorders. However, his mental status based on the Beck Anxiety Inventory (BAI) was 24, which belongs to mild anxiety disorder. Additionally, no mental disorder and cognition impairment occurred in this patient during the 6-month follow-up.

## 3. Discussion

The causes of POD can be classified as either predisposing or precipitating factors. It is often difficult to pinpoint a specific cause, and most of the time, it is the sum of predisposing and precipitating factors. Thus, we screened preoperative, intraoperative, and postoperative risk factors for pediatric ED and POD to discuss the potential predisposing and precipitating factors for this patient.

### 3.1. Preoperative Risk Factors

Although preschool age and advanced age are recognized as risk factors for pediatric ED and POD, respectively [3], ED did occur in young adults as reported in this case and other previous cases [7–10], indicating that ED can occur at any age.

Many patients may suffer different degrees of anxiety, tension, and other adverse emotions from surgery, life, and work [7]. These abnormalities can result in continuous mental stress, which affects their sleep quality [11] and the central nervous system, and are risk factors for POD. According to the analysis of the published ED case reports [7–10], most of the young patients had mental state abnormalities after medical history review. In our case, the patient had a mild anxiety disorder based on BAI, which may be the predisposing factor for this ED.

### 3.2. Intraoperative Risk Factors

Surgical type, duration, emergency surgery, blood loss, and transfusion are risk factors for POD [6]. Surgery duration greater than 3 hours in cardiac surgery or 6 hours in head and neck cancer resection [12] is an independent predictor of POD. In this case, the appendectomy was a small surgery with no obvious blood loss and was completed in 2 hours. Moreover, the case of POD or ED after appendectomy had never been reported in the literature.

Nearly most of the anesthetic drugs have been reported to be the risk factors of POD. Midazolam was implicated in a 26-month-old child who developed delirium following 0.5 mg/kg of an oral dose [13]. Corticosteroids have been reported to be a probable cause of delirium in a dose-dependent manner or under continuous use [14, 15]. Our patient received a small dose of midazolam and dexamethasone. Thus, they are unlikely to contribute to this ED. The effect of opioids is unclear, but studies found remifentanil [16] and sufentanil [17] could reduce the incidence of ED. As far as we know, no studies found that rocuronium is associated with POD. Thus, remifentanil, sufentanil, and rocuronium are unlikely to be risk factors for ED in this case.

Propofol and volatile anesthetics are commonly used anesthetic agents. Propofol had been implicated in delirium, hallucinations, and amorous behavior in the postoperative period [9]. A case reported a 20-year-old female patient who had ED after propofol anesthesia [8]. However, recent findings suggest that low-dose propofol sedation reduces the risk of delirium in the elderly [18]. Moreover, this patient received only 100 mg propofol for induction. Considering the short time of elimination and the small dose, propofol is an unlikely culprit.

Inhalational maintenance of anesthesia is a high-risk factor for pediatric ED, but in adults, it has not been frequently reported. The incidence of ED in adults following sevoflurane anesthesia reached 11.8% [19]. Compared with total intravenous anesthesia, inhalational anesthesia is associated with a higher rate of ED in adults [5, 20]. Thus, sevoflurane maintenance is likely to be the precipitating factor for this patient.

### 3.3. Postoperative Risk Factors

Length of intensive care unit (ICU) stay and postoperative pain were recommended as postoperative risk factors for POD [6]. Clearly, the length of ICU stay is not the cause of ED. In this case, a PCIA was used after surgery and the patient reported no pain during the perioperative period, indicating that postoperative pain is unlikely to be the cause of ED.

Taken together, in this case, the likely predisposing factor for ED is his worry about occupation mobility and the precipitating factor is inhalational maintenance with sevoflurane. However, what are the potential mechanisms of the ED occurrence caused by mental stress and inhalational maintenance with sevoflurance?. The most accepted theory holds that delirium is a neuropsychiatric manifestation caused by disturbances in brain metabolism and neurotransmitters [21]. Constant stress or anxiety may lead to hypersensitivity of neural communication. The higher incidence of ED after inhalational anesthesia than total intravenous anesthesia may be primarily related to the low blood solubility and “fast in and fast out” nature of inhalational agents [3, 22, 23]. Therefore, it is possible to cause nonsynchronous neural excitation and abnormal neural communication; as a result, ED occurs. Although no difference of recovery time between the inhalational anesthesia and total intravenous anesthesia was found [23], which seems inconsistent with our hypothesis, we cannot ignore that the “fast out” of inhalational agents is usually achieved by manually increasing the air flow as done in this case (8 L/min), while the intravenous anesthetics are automatically eliminated. To demonstrate this hypothesis, a study comparing high “wash-out” with low “wash-out” flow rate after sevoflurane anesthesia will be carried out in our hospital.

## 4. Conclusion

ED occurs at any age of patient and in any minor surgery. The unnoticeable mental stress may be the predisposing factor and the sevoflurane maintenance of anesthesia may be the precipitating factor for ED in young healthy patients who underwent minor surgery. Anesthesiologists should do some work to prevent it from happening.

## Data Availability

All data included in this study are available upon request by contact with the corresponding author.

## Supplementary Materials

Supplementary Materials The supplementary videos can be downloaded from the following link: https://pan.baidu.com/s/1BbWUIcFVdENwL3x5uSg1ZQ (code: 3tmm).
