# Supplementary material for: Emergence Delirium in a 29-Year-Old Man following an Uneventful Appendectomy
Source: Case Rep Med. 2021 Sep 25;2021:1338823. doi: 10.1155/2021/1338823 (PMC8487383; doi:10.1155/2021/1338823)
Supplement: Supplementary Materials — The supplementary videos can be downloaded from the following link: https://pan.baidu.com/s/1BbWUIcFVdENwL3x5uSg1ZQ (code: 3tmm). [file 1338823.f1.docx]

**Supplementary videos**

The supplementary videos can be downed from the following link:

<https://pan.baidu.com/s/1BbWUIcFVdENwL3x5uSg1ZQ>

Enter Code: 3tmm
